# Multidrug-Resistant Fungi

**DOI:** 10.3390/jof10100686

**Published:** 2024-09-30

**Authors:** Daniel Clemente de Moraes, Antônio Ferreira-Pereira

**Affiliations:** Laboratório de Bioquímica Microbiana, Departamento de Microbiologia Geral, Instituto de Microbiologia Paulo de Góes, Universidade Federal do Rio de Janeiro, Rio de Janeiro 21941-902, Brazil

Multidrug resistance in fungi is a growing challenge to global public health, resulting in ineffective treatments and thus high mortality rates. This phenomenon is boosted by several factors, such as the indiscriminate use of antifungals, the ability of fungi to evolve resistance mechanisms rapidly, and the dissemination of resistant strains through healthcare facilities and the overall environment. Understanding the molecular basis of resistance and the routes of dissemination of fungi is essential to allow the development of strategies that are able to treat and control resistant fungal infections.

In this Special Issue, eight original articles were published. Yadav et al. (2023) reported the isolation of *Candida auris* from the ears of four dogs in India [1]. All the strains presented high MIC values for fluconazole and were susceptible to other azoles, such as voriconazole and posaconazole, and to drugs belonging to different classes, such as amphotericin B, flucytosine, and echinocandins. According to the World Health Organization (WHO) fungal priority list, *C. auris* is a critical pathogen due to its intrinsic resistance to antifungal agents, the unacceptable mortality rates of its infection, and its ability to spread in hospitals. The study conducted by Yadav et al. described that animals could act as reservoirs for *C. auris*, which may suggest the possibility of interspecies transmission and reinforces the need for additional studies regarding the epidemiology of this fungus.

Casimiro-Ramos et al. (2024) assessed the genome of *C. auris* 20-1498, the first resistant *C. auris* isolated from Mexico [2]. This study detected a point mutation in ERG11, justifying this strain’s resistance to fluconazole. *C. auris* 20-1498 displayed thermo-tolerance and halotolerance, features that could facilitate its survival on several surfaces, such as the human body and healthcare facilities.

The study conducted by Parra et al. (2024) evaluated the expression of genes related to azole resistance in *C. glabrata* strains isolated from ICU Colombian patients and observed that exposure to fluconazole can downregulate or upregulate the expression of these genes [3]. In addition, docking analysis showed a low affinity of fluconazole to lanosterol 14-alpha-demethylase, which may explain the high degree of resistance of *C. glabrata* to this antifungal agent.

In order to better understand the adaptation of *Aspergillus fumigatus* to azoles, the study conducted by Hokken et al. (2023) investigated the transcriptional response of three *Aspergillus fumigatus* isolates to azole compounds, such as itraconazole and isavuconazole [4]. Understanding the genes and pathways involved in adaptation to azole compounds could lead to the creation of new antifungal drugs or the improvement of existing therapies, enabling overcoming azole resistance. The azoles tested in this study affected the expression of genes related to sterol metabolism, adhesion, efflux pumps, secondary metabolites, and stress response pathways. 

Lucio et al. (2024) assessed the relation between the Mismatch Repair Protein Msh6 and antifungal resistance in *Aspergillus fumigatus* [5]. Deletion of Msh6 led to a higher tendency for developing resistance to non-azole fungicides. The findings suggest that mutations in Msh6 may contribute to the multidrug resistance observed in clinical and environmental isolates of *A. fumigatus*. This knowledge is important for developing new strategies to manage resistant Aspergillus infections.

Three of the studies published in this Special Issue assessed the effect of different substances against resistant fungi. Using two *C. albicans* and one *C. glabrata* clinical isolates, Rollin-Pinheiro et al. (2023) verified the antifungal activity of the depsipeptide aureobasidin A [6]. This compound affected Candida planktonic cells and biofilms. It led to oxidative stress, diminished chitin content, and disturbed mitochondria, among other effects within the cells. Notably, aureobasidin A inhibited CaCdr2p and CaMdr1p in *Saccharomyces cerevisiae* mutant models. These are two of the three main efflux transporters related to the multidrug resistance phenotype in *C. albicans*. The effect of this inhibition was seen in an in vivo model, where the combination of aureobasidin A with fluconazole increased the survival of *Caenorhabditis elegans*.

Silva et al. (2023) developed a new synthetic route to produce Altissimacoumarin D. They tested the ability of this molecule and analogues to inhibit *Candida albicans* efflux pumps (CaCdr1p, CaCdr2p, and CaMdr1p), using *S. cerevisiae*-and *C. albicans*-resistant strains [7]. Two compounds (ACS47 and ACS50) inhibited ABC and MFS transporters, hence reversing the multidrug resistance phenotype. Moreover, they did not display toxicity in vitro and in silico. Therefore, these coumarins may be potential candidates for use in association with fluconazole to treat *C. albicans* azole-resistant infections.

In a drug repurposing study, Xisto et al. (2023) assessed the effect of miltefosine on Mucorales fungi, such as *Rhizopus oryzae* and *Mucor velutinosus* [8]. Miltefosine presented fungistatic activity, disrupted biofilms, and affected the cell wall content. Moreover, it likely interacts with fungal lipids. This study is a great example of the importance of drug repurposing to discover new antimicrobial drugs.

This Special Issue presents manuscripts that are collectively crucial to the field of antifungal resistance, covering the dissemination of resistant fungi, mechanisms of resistance, and the discovery of potential antifungal agents. We are grateful to the authors for their contributions to this Special Issue and to the study of resistance in fungi. These microorganisms are severely neglected, and it is through high-quality research that we can hope to overcome resistant fungal infections. We also extend our gratitude to the reviewers for their valuable corrections and suggestions.
